# Mr. Ellis's Biography of Dr. Gordon

**Published:** 1823-06-01

**Authors:** 


*Memoir of the Life and Writings of John Gordon, M.D. F.R.S.E. late Lecturer on Anatomy and Physiology in Edinburgh.* By Daniel Ellis, F.R.S. E. Edin. 1823. Pp. 238, Duodecimo.

The biography of medical men is but scanty, and the greater part of even that which exists, is not peculiarly interesting. The tenour of the lives of the generality of our profession is too even,—too little checkered by eventful circumstances to give zest and interest to a narrative; and its duties and anxieties are so uniform, and happen so alike to all, that one day may be said to tell unto another its unvaried history. In this deficiency of personal anecdote, and of variety in the scene of action, the biographer must of necessity draw his materials chiefly from the writings of the subject of his memoir, and his great aim must be, to unfold the circumstances which gave rise to, and assisted the progress of, his opinions, and to mark the influence which they exerted on the theories and practice of the times. A work of this nature, though, perhaps, not strictly conformable to the rules of regular biography, might undubitably be rendered very interesting, and, which is better, very useful, provided the person whose life and labours it recorded, was confessedly one of pre-eminent acquirements, and whose opinions had, in fact, influenced those of his professional brethren.* But if employed in the

* Such should be a life of Dr. Cullen, a memoir of whom has been read to the Royal Society of Edinburgh, by Dr. Thomson. As their Transactions are in the hands of few professional men, might we suggest to the Doctor the propriety of his publishing this work in a separate form?

eulogy of a person yet unknown to fame, or but partially so, whose talents were appreciated only by his friends, and those who had come in contact with him, and had not advanced him, without question, beyond the level of his compeers,—whose labours could bear comparison with theirs, and it should be doubtful to whom the palm was to be awarded,—if such were the case, the work could scarcely be expected to excite any general attention. And such appears to be the case with the late Dr. Gordon;—his fame was in its infancy, and he was taken away ere he had gained the eminence which seemed within his reach,—and we, consequently, doubt whether, beyond the circle of his friends and pupils, the work before us, so honourable to his memory, and to the feelings and taste of its author, will have any great circulation.

Dr. John Gordon was a younger son, and born in Forres, on the 19th of April, 1786. After receiving the rudiments of education, partly under his paternal roof, and partly in the grammar school of his native place, he was removed, towards the end of 1799, to the University of Edinburgh. Here he continued, for about two years, to prosecute his classical studies, after which time, he became an apprentice to Dr. Thomson, Professor of Surgery, and so advantageously known to the profession by his Lectures, and Researches into Syphilitic and Varioloid Diseases. By punctuality in the discharge of his duties, and diligence in study, he secured the approbation of his preceptor, who subsequently proved one of his best and most attached friends.

During his apprenticeship, Dr. Gordon, as is customary with young men in similar situations, attended the medical classes in the University, and those of the more distinguished private lecturers. In 1803, he was elected a member of the Royal Medical Society, in the proceedings and discussions of which, he "distinguished himself as well by his knowledge and ingenuity, as by the ease and gracefulness of his elocution." No notice is taken of the dissertations which he read before this body, though one of them, at least, was superior, we believe, to the generality, and rested its conclusions on original experiments. In this Essay, as we learn from the excellent Thesis of Dr. Cheyne, he anticipated Dr. Halliday in some of his most interesting statements, and was the first to collect the facts illustrative of the condition of the lungs in wounds of the thorax, and more particularly in emphysema.*

* It may be not unnecessary to quote the passage.—"In dissertatione Doctoris Gordon nuper defuncti, et tam merito defleti, facta hac de re primum collecta atque elucidata erant. Doctor Halliday laudem minime parvum obtinuit, ob explicationem factorum eorundem quam dedit. Sed

In the spring of 1805, Dr. G. obtained his diploma from the Royal College of Surgeons of Edinburgh, and on the 24th of June in the same year, he proceeded to the degree of Doctor in Medicine in the University. It was the intention of his friends that he should now enter the service of the East India Company, but, to this plan, Dr. G. was himself disinclined, and, at the suggestion of Dr. Thomson, resolved to become a Lecturer on Anatomy and Physiology. He began immediately to qualify himself, by dissection and study, to teach these branches of medical science; and the better to accomplish this object, he, in the winter of 1805, visited London, and became a pupil of the School in Great Windmill Street, then under the able direction of the late Mr. Wilson, from whose lectures, he acknowledges himself, to have derived not only profit, but pleasure.

On his return to Edinburgh, in the summer of 1806, he continued to prosecute, with unabated ardour, his favourite studies; and, at the commencement of the winter session, was elected one of the annual Presidents of the Medical Society. This was a circumstance peculiarly fortunate, since, here he acquired the friendship of his ingenious biographer, who was his associate in office, and who, even at that period, was engaged in those enquiries which have placed him in such a distinguished station amongst physiologists.

"The circumstance," says Mr. Ellis, "of my having cultivated a small portion of that field of science in which he exerted himself with so much ardour, served to increase the interest we took in each other's pursuits, and to multiply the occasions of bringing us together. He who has himself been engaged in the doubts and perplexities of original enquiry, can best estimate the value of a friend, ready, at all times, to hear with patience, and discuss with candour, the yet immature views presented to him;—to correct with delicacy what may be erroneous—with honesty to suggest what may be deficient—and zealous to approve that which may be just and true. Such was the friend I had found in Dr. Gordon, and such too, alas! is the one I have lost by his death." 15.

It was now time for Dr. G. to enter upon his public life. He had no expectations of any material assistance from his father, and was thrown, at the very outset, on the resources of his own talents and industry. During the summer of 1807, on the suggestion of Dr. Thomson, with whom he at this time resided, he gave some demonstrations in osteology to a few

---

haud incertum est eum copiose a dissertatione Doctoris Gordon mutuatum; et laus ei data quæ alteri debebatur, ostendit, eum non satis auxilium acceptum agnoscere."—*Diss. Med. Inaug. de Emphysemate, p.* 39, *Edinburgh,* 1820.

of his particular friends, who were soon satisfied that he possessed every qualification requisite in a teacher of anatomy. Encouraged by their approbation, he announced, in December of the same year, a course of public demonstrations, which was attended by a few pupils, the number of whom rapidly encreased. At first, Dr. G. taught anatomy and physiology in conjunction, but, from experience, having become convinced, that this plan could not be pursued with advantage to either of the sciences it professed to teach, he was induced, in the summer of 1813, to commence a separate course of lectures on the latter, which had now become his favourite study, and to which he devoted himself with great ardour. Those who estimate aright the extent of these branches of our art, may think with us, that the pages which the author has written in justification of this plan of Dr. Gordon's unnecessary. In these admirable lectures, he presented a fuller and more philosophical view of the science, than had, perhaps, ever before been given in this country. The plan, an account of which occupies a considerable portion of the volume, was his own, and framed, with great judgment, so as to bring under review, in not an unnatural succession, the varied topics of discussion. In discoursing on any function, he was accustomed, first to state the facts which had been ascertained;

" And when the inferences which arose coalesced into a series of propositions, and assumed the form of a theory or general law, it was exhibited in the plainest language, and with such reservations, doubts, and exceptions, as the state of our knowledge, or his own reflections, might suggest. Though always desirous of proceeding by the method of induction, yet, where that was impracticable, he did not wish to banish all hypothetical views from physiological investigation. He knew well, that a collection of mere facts, however complete, possesses, comparatively, little interest or value, unless an attempt be made to arrange and connect them, by the exercise of our reasoning powers: that, in the present auspicious period, when the human mind is so little disposed to yield to any authority save the convictions of its own reason, hypotheses produce few of the evils which they formerly engendered: and that, professing to rest, as they must now do, on facts derived from observation and experiment, they cannot maintain their ground when better experiments and observation shall conduct to juster views. Till this shall happen, they facilitate the apprehension and remembrance of facts; give to them an interest which they could not otherwise command; and, by exciting attempts either to verify or disprove them, suggest often new researches, by which new truths are disclosed, and the boundaries of science enlarged. Hypotheses such as these, Dr. G. did not refuse to entertain; but, he discarded from his lectures all those fanciful speculations, resting, not upon facts, nor even on rational analogies, but on a set of undefined and indefinable terms, invented to conceal ignorance and protect error, and, therefore, contributing so much more to extend the surface, than to increase the solidity of science." 118, 120.

As a lecturer, Dr. Gordon has been seldom equalled, and perhaps never surpassed. His personal appearance was very prepossessing, and no sooner had he entered the room than all was quietness and attention. His reputation as a teacher of anatomy was very high.

"He possessed," says his biographer, and the statement is nothing more than correct, "a neatness and dexterity of hand, which peculiarly qualified him for anatomical investigations, and a patience and perseverance in pursuit not easily to be tired out, or subdued by difficulties. During the winter session, he laboured incessantly in preparing the objects to be demonstrated in his lectures; and, by his manner of exhibiting and describing them, removed much of the disgust liable to be excited by attendance on an anatomical course. Without the smallest approach to unnecessary refinement, or affectation of seeming delicacy, he contrived to exhibit parts with such attention to neatness and propriety,* and delivered his demonstrations in a manner so clear and interesting, as to render his lectures not only instructive, but amusing." 212.

But his lectures on physiology were still more attractive, and afforded him a wider field for the display of his talents.

"With clear conceptions of his subject, and just and comprehensive views, he expressed himself in language so simple and precise, and with so much ease and grace, as to secure always the fixed attention of his audience, and not unfrequently suspend them in silent admiration. His elocution was uncommonly distinct and pleasing, possessing even a degree of musical harmony; and the elegance of manner, which he uniformly displayed, was not the result of study or of art, but the happy effort of good taste improving on the free gifts of Nature. Deeply impressed with the importance of the science he had undertaken to teach, and ardent in the study and improvement of it, he was anxious to communicate to his hearers, not the knowledge only which he possessed, but a portion of that real love for the science with which he was himself animated. His pupils came therefore to his class, not with the desire simply of being taught, but the expectation of being gratified—not merely to perform certain stated duties, and pass an hour in listless indifference, or perhaps settled disgust, but to acquire knowledge in a manner the most inviting, and bear away with them a love for the studies in which they had been so agreeably occupied." 214.

---

* In this respect in particular we must needs be allowed to say that he was a perfect contrast to lecturers on anatomy in general, and that they would do well to imitate his example.

If, as a lecturer, Dr. G. had a fault, it was what we must be allowed to term his over-scepticism. That in a science encumbered with so many "false facts" and foolish hypotheses as physiology, much caution and judgment in their selection is necessary, we readily allow, but in him we do think it was carried to a somewhat blameable excess. It is needless to particularize, for on almost every subject doubts were started, and the student was left in uncertainty. It may require, it is true, much more pains to make young minds perceive the difficulty, than to understand the solution of a question; but when objections are urged to every solution, they are apt to acquire the pernicious habit of hunting for a flaw in every train of argumentation, and of imagining some deficiency in every experiment; and more pleased when they can detect some such imaginary fault, than anxious to perceive the beauty of the one, or the aptness of the other.

From a desire to finish at once what we had to say of Dr. G. as a lecturer, we have strayed from the narrative, to which we now return to give some account of his literary labours. In October 1808, having been admitted a Fellow of the Royal College of Surgeons, he chose for the subject of his Probationary Essay the "Dislocations of the Thigh Bone." To verify the accuracy of the descriptions of others, and rectify the discordance that existed among them, he not only studied the cases recorded, but had recourse to experiments on the dead body.

"To discover what muscles contributed to retain the head of the bone in its displaced situation, and what were most calculated either to oppose or to favour its return, Dr. G. produced artificially, on the dead body, the several varieties of luxated femur that occur in practice. Previously, however, to these operations, the soft parts surrounding the articulation were exposed by dissection; so that he might be able to mark at once the efficiency of certain parts in resisting luxation, the position which the head of the bone occupied when the luxation had taken place, and the derangement induced among the muscles. So correctly," we are told, "did this artificial imitation correspond with the dislocations that occur in the living subject, that it was found exceedingly difficult, if not altogether impossible, to produce artificially any other species of dislocation than those which actually present themselves in practice; and the external character of the real accident in the living body, and of its imitation in the dead one, always corresponded closely with each other." 19.

But we cannot enter into any detail of the information thus gained:—suffice it to say, that the Essay seems highly creditable to his talents and ingenuity.

Dr. G. was elected a member of the Royal Society of Edinburgh in January 1812, and he soon afterwards communicated some very interesting observations on the case of James Mitchell, a boy born blind and deaf, which are published in the Transactions of that body. But the principal work of Dr. G. is his "System of Human Anatomy." In 1815 the first volume was given to the public, and though the greater part of the materials of the subsequent volumes were collected, yet he lived to finish no more of his original design. It was his intention, in this work, to exhibit not only a full view of the science as taught in this country, but also to give the latest discoveries and improvements of the continental anatomists. The arrangement adopted seems entitled to great praise, and nothing can exceed the general perspicuity and conciseness of its descriptive language.

"He uniformly addresses himself to the eye, avoids all discussion of questions of doubt and opinion, and enters into minute details with a clearness and precision which those only who write from actual observation can command." 33.

His account of the structure of the skin may be specified as one of the most interesting pieces of descriptive anatomy extant; and that of the brain and nervous system is very elaborately written. His minuteness and precision may be ridiculed by those who make it their chief boast, and regard it as the highest excellence of the anatomist, to be able to cut at once upon this ramulus of an artery, or that ramusculus of a nerve; but they who are convinced that anatomy must be the principal guide of the physiologist in his disquisitions, will justly mark them as his chiefest excellencies. Of the work itself we shall add no further account, as it has been for many years before the public, to whom, we should hope, its merits require not now to be stated.

The next work of an anatomical nature from the pen of Dr. G. was called forth by the claims of Gall and Spurzheim. Perhaps no one in this country, from his previous studies and inquiries, was better entitled to give an opinion upon the subject; and looking upon the whole doctrines taught by these gentlemen "as a piece of thorough quackery from beginning to end," he expressed that opinion in a noted, but too severe article on the subject, in the Edinburgh Review.

Mr. Ellis has urged several reasons to rebut the charge of undue severity or enmity which that article is said to breathe; and the treatment which Dr. G. subsequently received from Dr. Spurzheim, when the latter visited Edinburgh, was not calculated, he thinks, to make the former regret that he was its author. Mr. Ellis gives a succinct historical account of the anatomical discoveries of Gall and Spurzheim, and afterwards a copious analysis of Dr. Gordon's "Observations on the Structure of the Brain," comprising an estimate of their discoveries. In this work he is said, by Mr. Ellis, to have *demonstrated* that their methods of dissection were not original and not preferable to those more commonly followed; that their physiological principles were very questionable, and had seduced them to describe as facts what was only conjecture; that they had committed many errors in their anatomical descriptions, and had even described some parts which had nothing but an imaginary existence; and lastly, that the greater number of their supposed discoveries which were real, had been long known to anatomists. This very learned Essay, we are told, procured for its author the thanks of several philosophers and anatomists, and among these, of Cuvier, of whose approbation any man might be proud. It drew from Dr. S. a pamphlet designed as a reply, not to Dr. G. only, but to his other opponents in Britain.

"As an answer to my Treatise," said Dr. G. addressing his class, "I cannot regard it. It seems intended to be read only by those who have not perused my 'Observations.' It attributes to me opinions respecting both the functions and the structure of the brain, which I have no where expressed, either in my lectures or books. My objections were directly and pointedly urged; and they ought to have been as directly replied to; but, instead of this, the writer studiously evades them all." 88.

We believe, however, that all liberal-minded men on this side of the Tweed, agree in censuring the tone of Dr. Gordon's article in the Edinburgh Review, and in acknowledging that the mode of dissecting the brain adopted by Gall and Spurzheim is infinitely superior to that in general use.

The last work of Dr. Gordon's was a fasciculus of plates in octavo, exhibiting a series of representations of those parts of the human skeleton, with which it is of most importance for the physiologist and medical practitioner to be accurately acquainted. Of this work it may be sufficient to quote, in proof of its excellence, the opinion of Dr. Baillie. "The engravings," says that distinguished physician in a letter to the author, "are extremely well executed, the various sections well imagined, and the short descriptions possess all that precision for which you are distinguished." 103.

Besides these, Dr. Gordon was the author of several essays in various periodical publications, which are not noticed in the life before us, and which we may therefore be excused for passing over in equal silence. Engaged in the pursuits of anatomy and physiology, Dr. G. had at first declined all thoughts of engaging in medical practice; but a few years before his death, having mastered most of the difficulties which the earlier stages of his life presented, and his duties, as a teacher, having become comparatively easy, he did not refuse to enter upon its duties; and his worth of character, and amenity of manners, introduced him, without effort or solicitation, to a considerable share of the most respectable practice. Thus occupied, and with the prospect of "honours and dignities" before him, he was, in the commencement of June 1818, attacked with that disease which, on the 14th of the same month, terminated his earthly career.

Dr. G. was married in 1812 to Miss Rutherford, between whom and himself a mutual esteem and affection had long existed. By this lady he had four children, of whom one son and two daughters survive their father. In his family, and in the circle of his friends, he was exceedingly beloved.

> "The cheerfulness of his nature—the equal and happy balance of all the powers of his understanding—the large range and variety of his accomplishments, and the sweet pliability of his spirits, fitted him, in an eminent degree, for all the pleasures of society; and, while he reserved his love and confidence for the generous and intelligent, who formed the inner circle of his friends, he had the utmost indulgence for all the ordinary defects and infirmities, and lived easily and kindly with the whole range of his acquaintances." 208.

Nothing could better evidence the estimation in which he was held, than the strong sensation which his death produced, and which was altogether unprecedented in that of a private individual, whose merit was his only claim to public favour. A large portion of the genius and learning of the northern metropolis, as well of the other learned professions as of that to which he belonged, honoured his obsequies by their presence:—but among the persons who attended this solemn ceremony, none bestowed on it greater interest than the members of the Royal Medical and Physical Societies, to which belong the greater number of senior students who frequent the University. On the mournful occasion of his death, they assembled in their respective halls, and passed resolutions expressive of their admiration of his talents, their sorrow for his loss, and their desire of testifying respect for his character by accompanying his remains to the grave.

Such is the brief analysis of this little work which our limits will permit, but it seems sufficient to give our readers a tolerable idea of what interest it is calculated to afford. It is, as we have already said, highly creditable to the feelings and good taste of its author, equally free from the affectation of morbid sensibility, and of overweening and indiscriminating partiality; and yet it can scarcely be expected that the profession in general will estimate so highly as he has done the talents of its subject, since that estimate is founded partly on a calculation of what might have been effected; and partly on grounds of which a friend only can be a judge. To most, we fear, the affecting lines of the poet will appear not altogether inapplicable.

> "He now is dead, and all his glory gone,
> "And all his greatness vapoured to nought,
> "That as a glass upon the water shone,
> "Which vanisht quite so soon as it was sought;
> "His name is worn already out of thought."
>
> SPENCER.

